# A Hybrid-Frequency Sampling Tactile Sensing System Based on a Flexible Piezoresistive Sensor Array: Design and Dynamic Loading Validation

**DOI:** 10.3390/s26051559

**Published:** 2026-03-02

**Authors:** Zhenxing Wang, Xuan Dou

**Affiliations:** 1 School of Computer and Information Engineering, Shanghai Polytechnic University, Shanghai 201209, China; 2 School of Intelligent Manufacturing and Control Engineering, Shanghai Polytechnic University, Shanghai 201209, China; 20241513122@sspu.edu.cn

**Keywords:** electronic skin, tactile array, Velostat sensor, dynamic tactile perception

## Abstract

**Highlights:**

**What are the main findings?**

- It was observed that although Velostat exhibits inevitable temporal drift in absolute resistance, the relative variation in channel responses is sufficiently stable to identify tactile states.
- System-level validation, supported by RTL simulation, confirmed that the hybrid-frequency scheduler executes deterministic scanning with an aggregate bandwidth of 36.9 kHz. The system exhibits high repeatability (RSD < 4.9%), strong linearity, and robust mechanical durability under cyclic bending.

**What are the implications of the main findings?**

- These results highlight a practical route toward FPGA-based dynamic tactile sensing systems that can capture fast-evolving contact behaviors with zero latency, which are typically missed by conventional e-skins.
- The work provides experimental evidence that relative-dynamics-based interpretation combined with deterministic hardware timing can mitigate material drift and system latency issues, offering new insight for the design of future soft tactile interfaces.

**Abstract:**

A Hybrid-Frequency Sampling Tactile Sensing System Based on a Flexible Piezoresistive Sensor Array is presented for reliable and real-time tactile perception under dynamic loading conditions. While recent studies have developed multi-channel tactile arrays, most systems remain limited by time-dependent drift in channel responses, inconsistent dynamic behavior, or insufficient temporal resolution under simultaneous loading. In this work, a system-level design integrating a flexible piezoresistive sensor array with a real-time data acquisition module is developed, incorporating a hybrid-frequency sampling strategy to reduce system complexity while preserving reliable dynamic response in key sensing channels. Register-Transfer Level (RTL) simulation verified that the hardware scheduler rigorously executed the deterministic scanning logic, demonstrating a strict one-to-one correspondence with the physical hardware signals. The array consists of 34 piezoresistive sensing nodes embedded in an elastomeric substrate. Under the implemented hybrid-frequency sampling scheme, the system achieves an overall effective acquisition bandwidth of approximately 36.9 kHz, while maintaining a repeatability better than 4.9% and robust mechanical durability under cyclic bending deformation. Dynamic loading validation was performed using a self-developed pressure comparison platform for measuring the normal contact force applied on the tactile surface, serving as ground-truth data to verify that the voltages acquired by the proposed system accurately correspond to the actual applied force. Quantitative analysis shows a strong linear correlation (R^2^ ≈ 0.98) between the e-skin outputs and the reference forces. The recorded responses exhibit clear intensity-dependent trends and good temporal correspondence among sensing nodes, successfully distinguishing tactile stimuli such as gentle tapping, moderate pressing, and firm contact. The system also captures dynamic tactile responses during finger stroking, showing characteristic multi-unit activation patterns under spatiotemporally varying contact conditions. Compared with previously reported tactile systems typically operating below 100 Hz, the proposed design achieves an approximately 10× enhancement in effective sampling capability while significantly reducing system complexity through hybrid-frequency sampling, thereby supporting reliable dynamic tactile sensing in multi-unit arrays. These results demonstrate that the proposed system provides a practical and scalable hardware platform for dynamic tactile sensing in robotics, human–machine interaction, and wearable tactile systems.

## 1. Introduction

Tactile sensing is a key technology for robotics, human–machine interaction, wearable electronics, and intelligent sensing systems, as it provides direct information about physical contact, interaction forces, and surface properties. In robotic systems, tactile feedback plays an essential role in contact detection, force regulation, compliant control, and safe physical interaction. In wearable and healthcare applications, tactile sensors enable continuous pressure monitoring, motion tracking, and long-term physiological signal acquisition [1]. To support these emerging applications, tactile sensing systems are increasingly required to be flexible, conformable, and capable of distributed multi-point perception [2]. With the rapid development of flexible electronics and advanced functional materials, electronic skin (e-skin) systems with distributed and multi-point tactile sensing capability have attracted increasing research attention. While flexible piezoresistive sensors offer significant advantages in conformability and scalability compared to rigid counterparts, their practical application in large-scale dynamic sensing is often limited by system-level bottlenecks rather than material properties [3,4,5]. By integrating multiple piezoresistive sensing units into array configurations, multi-channel tactile sensing systems have been demonstrated for applications such as robotic manipulation, physical human–machine interaction, and wearable health monitoring [6,7]. These tactile arrays enable spatially resolved pressure measurement and allow distributed tactile information to be captured over extended contact regions. Specifically, ensuring high spatiotemporal resolution across a multi-unit flexible array presents distinct challenges for the data acquisition interface. Conventional time-division multiplexing (TDM) scanning methods often suffer from inter-channel time skew, causing signal distortion when capturing rapid dynamic events like sliding or impact. Conversely, fully parallel acquisition architectures can solve synchronization issues but incur prohibitive hardware complexity and power consumption when the number of sensing nodes increases. Therefore, developing a specialized acquisition architecture that balances system complexity with dynamic tracking capability is as critical as the sensor device design itself [8]. However, as the number of sensing units and the effective sensing area continue to increase, system-level challenges become increasingly prominent, particularly in terms of inter-channel signal coupling and time-dependent drift of the sensor baseline resistance, as well as maintaining reliable operation under dynamic loading conditions [9].

For dynamic tactile sensing, the overall system architecture plays a decisive role in determining whether fast and spatially distributed contact processes can be faithfully captured. In multi-unit tactile arrays, the ability to acquire signals from all sensing units in real time and in a synchronized manner is fundamental for system-level dynamic measurements. To address these requirements, various hardware- and software-based acquisition strategies have been reported, including time-division multiplexed scanning architectures, parallel acquisition, etc. Multiplexed scanning architectures can reduce hardware complexity, but are often limited by sampling rate and channel-to-channel timing skew [10,11]. In contrast, fully parallel acquisition architectures incur substantial system overhead, making them less suitable for large-scale tactile arrays. To address the trade-off between high-frequency feature extraction and data transmission bandwidth, we propose a ‘Hybrid Frequency Sampling’ architecture. Conceptually, ‘Hybrid’ refers to the heterogeneous allocation of sampling rates matched to the signal bandwidth of different sensing modalities. Technically, this is implemented via a deterministic sequencer based on a static Look-Up Table (LUT) embedded in the FPGA state machine. In contrast to dynamic priority scheduling, our system utilizes a fixed, pre-defined scanning sequence to enforce precise timing:

1. Oversampled Slots: Acoustic channels are explicitly mapped to multiple fixed indices within the sequence (e.g., appearing twice per cycle) to achieve the required 2 kHz rate.
2. Standard Slots: Dynamic tactile, acceleration, and environmental channels are assigned single fixed time slots within the sequence, resulting in a 1 kHz rate.
3. Sub-sampled Slots: Quasi-static monitoring channels are scheduled using a decimated frame counter logic (triggered once every 10 cycles) to maintain a 100 Hz rate, thereby optimizing system resources [12,13].

In addition to system design, experimental evaluation under dynamic loading conditions is essential for assessing the practical performance of tactile sensing systems. Many previous studies rely mainly on simplified loading methods, such as quasi-static indentation, step loading, or single-point cyclic compression, to characterize basic sensor performance including sensitivity, linearity, hysteresis, and repeatability. While these approaches are effective for device-level characterization, they do not fully represent realistic contact conditions encountered in practical robotic interaction and wearable applications, where tactile sensors are often subjected to time-varying forces, spatially distributed contacts, and multi-point dynamic loading [14,15]. Therefore, dynamic loading experiments at the system level are necessary to evaluate the capability of multi-unit tactile sensing systems to track time-varying contact processes. These time-varying contact processes include not only normal loading at fixed locations, but also realistic interaction patterns such as finger stroking, where the normal contact force distribution moves across a local region of the tactile surface over time.

To address these system-level gaps, this study shifts the focus from material synthesis to system-level integration and dynamic validation. We present a multi-unit tactile sensing system based on a flexible piezoresistive sensor array, in which a hybrid-frequency sampling strategy is implemented to reduce system complexity while preserving reliable dynamic response in key sensing channels. Specifically, the timing determinism of the hybrid scheduler is explicitly verified via hardware logic simulation, and the mechanical robustness of the flexible circuit is validated through cyclic bending tests [16]. Comprehensive system-level validation is conducted under quantitative dynamic loading conditions. The proposed system consists of a 34-channel flexible tactile array mounted on a 7 cm × 4 cm flexible printed circuit board (PCB), with each sensing unit measuring 5 mm × 7.7 mm. An FPGA-based data acquisition architecture is employed to implement a hybrid-frequency sampling scheme, enabling different sensing channels to operate at appropriate sampling rates (e.g., 2 kHz, 1 kHz, and 100 Hz) according to their dynamic sensing requirements, thereby providing a stable hardware foundation for multi-unit dynamic tactile sensing. From a system design perspective, these specifications were determined to satisfy the critical requirements of potential robotic applications. The 1 kHz sampling rate was selected to satisfy the bandwidth requirements for real-time feedback loops (typically 1 ms), ensuring the system’s hardware capability to capture transient contact events. The sensing unit dimensions (5 mm × 7.7 mm) were selected to achieve a spatial resolution compatible with anthropomorphic grasping scales, balancing wiring complexity with sufficient contact area coverage. Furthermore, the hybrid-frequency architecture is a purpose-built design choice to minimize data redundancy while preserving high-fidelity acquisition for the most dynamic tactile channels. Thus, the system is positioned as a specialized sensing infrastructure optimized for dynamic interaction tasks.

To evaluate the dynamic response characteristics of the proposed tactile sensing system, synchronized comparative experiments under dynamic loading conditions were conducted using external reference force sensors [17]. Dynamic tests including light tapping, moderate pressing, and firm contact were applied to investigate the system-level response behavior across different loading intensities. Through these experiments, the ability of the proposed tactile array to track time-varying contact processes and reflect dynamic loading characteristics was systematically evaluated.

It should be emphasized that the focus of this work is on the system-level design and dynamic validation of a multi-unit tactile sensing platform at the physical sensing level, in which a hybrid-frequency sampling strategy is employed to balance system complexity and dynamic sensing performance. Although this platform is motivated by the long-term development of embodied intelligence and future physical human–robot interaction, the present study does not address higher-level perception, cognitive modeling, or interaction strategies. Instead, the sensing system plays a critical role as the fundamental physical interface that bridges environmental stimuli and computational intelligence. By providing high-fidelity, synchronized, and time-resolved tactile data streams, it establishes the necessary low-level sensory infrastructure required to support the development of robust perception algorithms and complex interaction strategies in future work.

Overall, the proposed multi-unit tactile sensing system offers a reliable and scalable hardware platform for high-performance tactile perception under dynamic loading conditions. By combining a flexible piezoresistive sensor array with FPGA-based parallel acquisition and system-level dynamic validation, this work addresses key challenges in multi-channel tactile sensing and provides a practical experimental foundation for future research in intelligent tactile sensing systems, robotics, human–machine interaction, and wearable tactile technologies [1,18,19].

## 2. Materials and Methods

**Figure 1:**
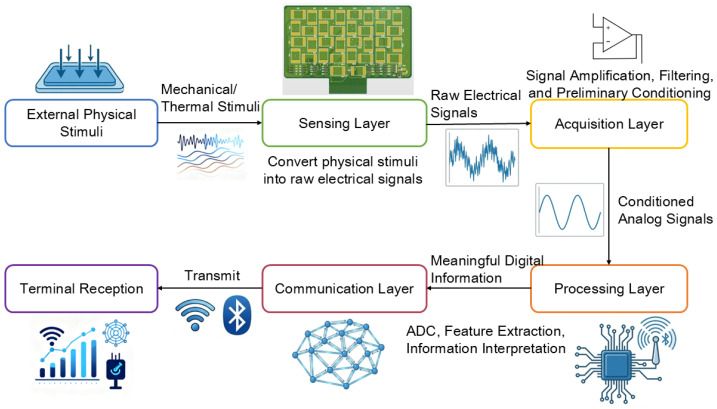
Overall architecture of the multimodal e-skin sensing and acquisition system.

The overall workflow of the proposed multimodal e-skin sensing and acquisition system is shown in Figure 1.

### 2.1. Flexible Tactile Array Design

The e-skin system is implemented on two flexible FPC layers: one sensor board accommodating all sensing units, and one acquisition board integrating the analog front-end and digital processing circuits [20].

**Figure 2:**
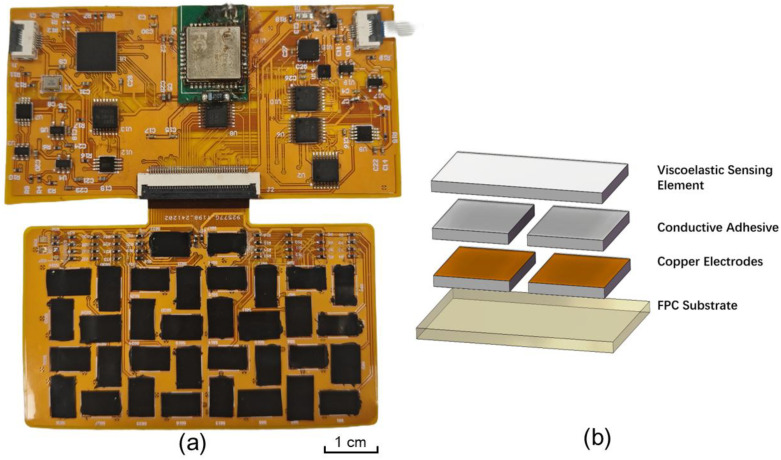
Fabrication and structure of the flexible tactile sensing system. (**a**) High-resolution optical photograph of the fully integrated system fabricated on a flexible polyimide (PI) substrate. The **upper section** shows the data acquisition board integrated with key surface-mount components, including the microcontroller, FPGA, analog multiplexers, and the wireless transmission module. The **lower section** displays the 34-channel tactile array, where individual laser-cut Velostat piezoresistive elements are bonded onto the FPC electrodes using conductive adhesive. A scale bar (1 cm) is provided for dimensional reference. (**b**) Exploded schematic view illustrating the layer-by-layer composition of a single sensing unit, comprising the viscoelastic sensing layer, anisotropic conductive adhesive, interdigitated copper electrodes, and the base FPC substrate.

The tactile array is implemented on a 7 cm × 4 cm flexible printed circuit (FPC) that serves as both the mechanical support and the electrical interconnect layer (Figure 2). A total of 34 tactile sensing units is distributed over the active area. Each unit occupies approximately 5 mm × 7.7 mm and the gaps between neighboring units are about 1.27 mm, providing a relatively high spatial resolution over the sensing surface.

The overall system fabrication utilizes standard flexible printed circuit (FPC) manufacturing processes on polyimide (PI) substrates to ensure mechanical conformability. The sensor array fabrication involves three main steps:

Substrate Preparation: The base layer is a custom-designed FPC with interdigitated copper electrodes (ENIG surface finish) to prevent oxidation.

Sensing Element Integration: The pressure-sensitive elements are precision-cut from a viscoelastic piezoresistive film (Velostat, 3M, St. Paul, MN, USA) using a laser cutter to match the 5 mm × 7.7 mm pad dimensions.

Bonding Assembly: These piezoresistive sheets are bonded onto the electrodes using anisotropic conductive adhesive. The adhesive is cured under controlled pressure to establish stable electrical contact while maintaining the structural flexibility of the array.

Similarly, the data acquisition board is fabricated on a separate FPC substrate using surface-mount technology (SMT). Key components, including the TMUX1108PWR analog multiplexers (Texas Instruments, Dallas, TX, USA) and AD7940 14-bit ADCs (Analog Devices, Wilmington, MA, USA), are integrated via a low-temperature reflow soldering process to prevent substrate deformation. The two boards are interconnected via a high-density FPC connector.

When external pressure is applied, the contact condition and internal structure of the viscoelastic material change, leading to a measurable variation in resistance between the two pads. This piezoresistive behavior can be described by the relationship between the electrical resistance change and the applied normal pressure [21,22]. Within the linear working region (experimentally verified as 0–5 N in Section 3.3), the relative resistance change is strictly proportional to the applied normal force *P*, which can be expressed as:

(1) $$\frac{\Delta R}{R_{0}}=\frac{R_{0}-R_{p}}{R_{0}}\approx S\cdot P,$$

where $R_{0}\;$ represents the initial resistance of the sensing unit in the unloaded state, $R_{p}$ is the resistance under applied pressure, and S denotes the sensitivity coefficient of the viscoelastic material structure. Beyond this linear region, the sensor exhibits a monotonic but nonlinear response up to 10 N (defined as the effective measurement range) due to material saturation, as quantitatively demonstrated in Section 3.3. In this way, each unit functions as an independent pressure-sensitive element that can be addressed through the FPC routing and connected to the subsequent analog front-end and multiplexed acquisition circuitry.

### 2.2. Piezoresistive Signal Readout and Analog Front-End

**Figure 3:**
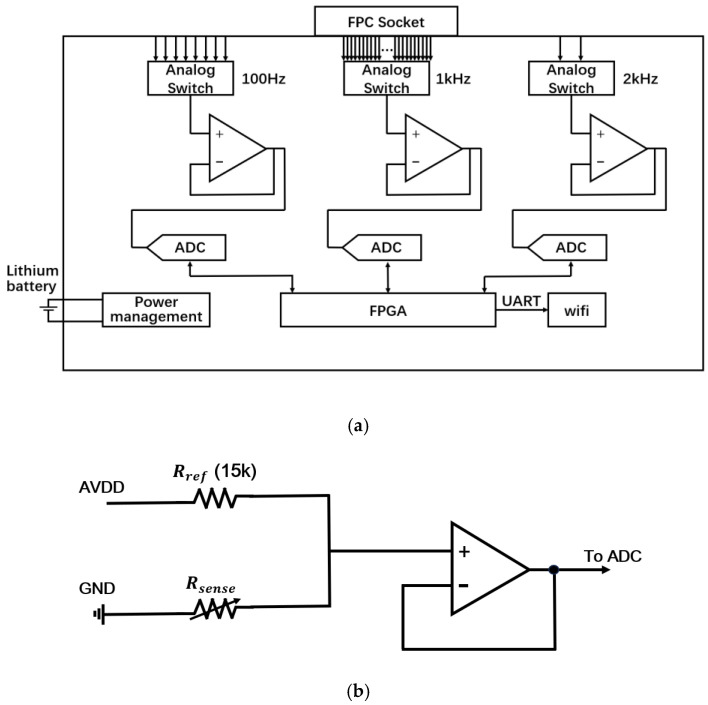
(**a**) Hardware architecture of the multi-rate data acquisition system. The system integrates analog switches, buffering amplifiers, ADCs, FPGA timing control, and wireless transmission modules to enable synchronized sampling of multi-modal signals. (**b**) Detailed schematic of the analog front-end circuit for a single tactile channel. The circuit employs a pull-up voltage divider configuration with a fixed reference resistor ($R_{ref}$ = 15 kΩ) and a unity-gain buffer (voltage follower) to isolate the sensor impedance before ADC sampling.

The resistance variation in each piezoresistive tactile unit is converted into a corresponding analog voltage signal through a voltage-divider circuit [23]. As illustrated in the detailed schematic in Figure 3b, the analog front-end utilizes a pull-up configuration where a fixed reference resistor ($R_{ref}$ = 15 kΩ) is connected between the analog supply voltage $V_{CC}$ and the output node, while the piezoresistive sensing unit ($R_{sense}$) connects the output node to the ground. According to Ohm’s law, the output voltage $V_{out}$ is determined by:

(2) $$V_{out}=\;V_{CC}\times \;\frac{R_{sense}}{R_{sense}+R_{ref}}$$

Consequently, an increase in sensor resistance (induced by applied pressure) results in a monotonic increase in the output voltage [24,25]. To ensure signal integrity, this voltage is buffered by a unity-gain operational amplifier (voltage follower) before being fed into the ADC. This buffering stage is critical to isolate the voltage-divider node from the input impedance of the ADC, thereby minimizing loading effects and ensuring accurate signal transmission without additional amplification [26].

Three AD7940 14-bit Successive Approximation Register (SAR) ADCs were used to handle the hybrid-frequency signals from the tactile, acoustic, and environmental sensing branches.

### 2.3. FPGA-Based Hybrid-Frequency Acquisition System

The hardware implementation of the multi-rate acquisition subsystem is illustrated in Figure 3.

As illustrated in Figure 3, the multimodal e-skin system adopts an FPGA-based multi-rate multiplexed acquisition architecture. All sensing signals on the flexible sensor board are routed to the acquisition board through an FPC socket. Three parallel acquisition branches are implemented to support heterogeneous sensing modalities operating at different sampling frequencies [27].

In each acquisition branch, a multi-channel analog multiplexer is used for channel selection, followed by signal buffering and an independent analog-to-digital converter (ADC). The three branches operate at 2 kHz for acoustic (vibration) channels, 1 kHz for tactile, acceleration, and environmental channels, and 100 Hz for quasi-static force channels, respectively. This parallel multi-branch structure enables each sensing modality to be sampled at its appropriate temporal resolution without mutual interference. The selection of these specific sampling frequencies is based on the distinct physical bandwidth requirements and synchronization needs of each sensing modality. The 2 kHz rate for the acoustic channels satisfies the Nyquist criterion for capturing high-frequency texture-induced vibrations (typically <1 kHz). The 1 kHz rate for the tactile, acceleration, and environmental channels is chosen to align with the typical 1 ms control cycle of robotic feedback loops, ensuring real-time responsiveness and strict synchronization between kinematic and tactile events. Finally, the 100 Hz rate is sufficient for the quasi-static force sensors given the slow dynamic nature of stable grasping, thereby minimizing data redundancy. The FPGA serves as the central timing, control, and synchronization unit of the acquisition system. It generates the channel-selection signals for all analog multiplexers, provides sampling clocks for the ADCs, and merges the digitized data streams into a unified time-ordered output. The merged data are then transmitted to a wireless communication module through a serial interface for real-time data streaming to the host computer.

**Figure 4:**
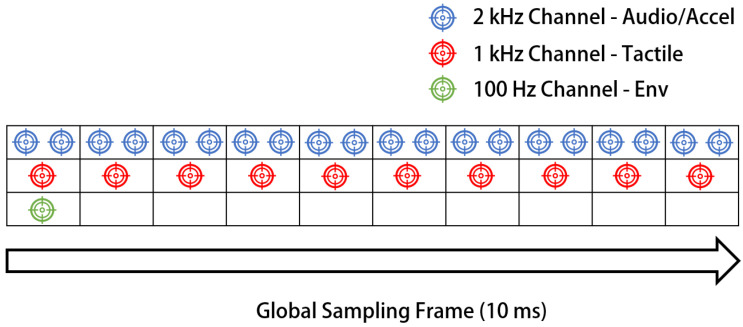
Timing diagram of the FPGA-based weighted round-robin scheduling scheme within a 10 ms global frame. The 2 kHz channels are sampled twice per 1 ms slice, the 1 kHz tactile channels once per slice, and the 100 Hz channels once per frame, ensuring synchronized data acquisition.

To maintain temporal consistency among heterogeneous sensing modalities operating at different sampling frequencies, a global-frame time-slicing scheduling mechanism is implemented in the FPGA. A 10 ms global sampling frame is defined and uniformly divided into ten 1 ms time slices. Each sensing channel is assigned one or more fixed sampling slots within this frame according to its designated sampling rate. Specifically, the 2 kHz acoustic channels are sampled twice within each 1 ms slice, the 1 kHz tactile, acceleration, and environmental channels are sampled once per slice, and the 100 Hz quasi-static force channels are sampled once within each 10 ms global frame. This deterministic scheduling strategy avoids sampling conflicts, preserves phase alignment among multi-rate channels, and ensures fully synchronized hybrid-frequency acquisition at the system level (Figure 4). The deterministic timing control of the FPGA ensures that the effective sampling rate for the multi-channel tactile array is strictly defined by the hybrid-frequency scheduling scheme. The per-channel sampling rate $f_{s}$ is determined by the global frame structure:

(3) $$\;f_{s}=\frac{1}{N\cdot T_{slot}+T_{overhead}}\;,$$

where N is the total number of tactile sensing channels, $T_{slot}$ is the time slot allocated for sampling each individual channel, and $T_{overhead}$ represents the minimal timing overhead reserved for channel switching and data synchronization. In this system, different tactile channels operate at different sampling frequencies, forming a hybrid-frequency acquisition scheme tailored to their respective dynamic sensing requirements. It should be noted that, although the acquisition system supports multiple sensing modalities and sampling rates, the experimental investigations reported in this paper focus exclusively on the tactile sensing branch operating at 1 kHz, which corresponds to the multi-unit flexible tactile array used for dynamic tactile evaluation.

To explicitly address the heterogeneous bandwidth requirements of the multimodal sensor array, the hybrid-frequency sampling strategy is realized through a Weighted Round-Robin (WRR) scheduler instantiated in the FPGA’s Finite State Machine (FSM). Unlike software-based scheduling, this hardware-level implementation ensures deterministic timing control with zero jitter.

The core of this scheduler is a pre-configured Look-Up Table (LUT) that maps time slots within a global scanning frame to specific physical channels. As detailed in Table 1, the system allocates sampling bandwidth based on three priority levels:

As shown in Table 1, the configuration logic ensures that different sensing modalities operate at their optimal temporal resolutions:

High-Priority Acoustic Slots (2 kHz): The two vibration sensing channels (CH35, CH36) are explicitly mapped to multiple discrete indices (e.g., Slots 0, 1 and re-entry at Slots 15, 16) within a single 1 ms scanning superframe. This interleaved “multiple-entry” mechanism physically enforces a 2 kHz effective sampling rate, satisfying the Nyquist criterion for high-frequency texture features.

Standard Multimodal Slots (1 kHz): The majority of the bandwidth is allocated to the “Standard” priority group, which includes 25 dynamic tactile units (CH1–CH25) and 5 auxiliary multimodal channels (3-axis acceleration, temperature, and humidity). These channels are triggered once per cycle, ensuring that kinematic and environmental data are strictly synchronized with the dynamic tactile events at 1 kHz.

Low-Priority Static Slots (100 Hz): For the 9 peripheral channels (CH26–CH34) monitoring quasi-static grasping forces, a secondary sub-sampling logic is applied. These channels share a limited number of time slots and are triggered in a rotational manner across ten consecutive frames, resulting in a highly efficient 100 Hz rate that reduces data redundancy by 90%.

### 2.4. External Reference Platform and Dynamic Loading Method

**Figure 5:**
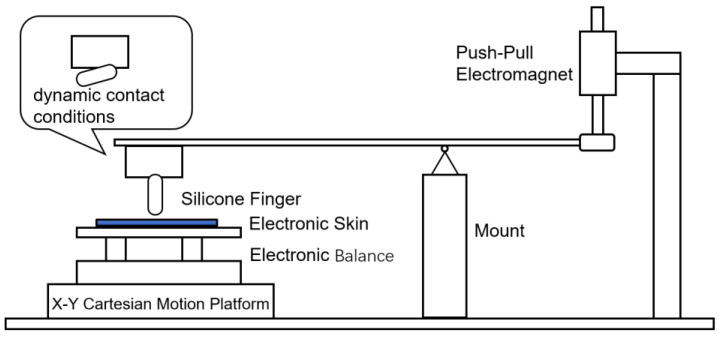
External reference platform for dynamic tactile loading and force measurement.

An external reference platform was constructed to provide controlled dynamic loading and independent force measurement for the evaluation of the proposed tactile sensing system. As illustrated in Figure 5, the platform consists of a silicone finger, an electronic Balance, an X–Y Cartesian motion stage, and a push–pull electromagnet integrated with a lever mechanism.

The silicone finger is used as a compliant contact interface to simulate soft fingertip-like interactions with the flexible tactile array. The tactile sensor is placed directly on top of an electronic Balance, which serves as an external reference sensor for measuring the applied normal force. The X–Y Cartesian motion platform is used to drive the electronic Balance and the tactile sensor to generate controlled planar motion, enabling the normal contact region to move continuously across the tactile surface under dynamic contact conditions.

A push–pull electromagnet mounted at one end of the lever structure provides controllable normal loading. By adjusting the actuation signal applied to the electromagnet, the silicone finger can be driven to press against or release from the tactile surface with repeatable force profiles. The lever mechanism ensures stable transmission of the electromagnetic actuation force to the contact point.

During experiments, dynamic tactile stimuli are generated through the combined action of vertical loading from the electromagnet motion from the X–Y platform. The vertical loading from the electromagnet provides the normal contact force, while the X–Y platform introduces controlled bidirectional in-plane motion, resulting in a spatiotemporally moving normal-force distribution on the tactile array. The normal force applied to the tactile array is simultaneously recorded by the electronic Balance and used as a reference signal for comparison with the outputs of the tactile sensing system. This setup enables synchronized acquisition of tactile responses and external force measurements under repeatable and well-controlled dynamic loading conditions.

### 2.5. Data Processing and Evaluation Metrics

The digitized tactile signals from all sensing channels and the external reference force measured by the electronic Balance are transmitted to the host computer for offline processing. All acquired data streams are first temporally aligned according to their time stamps to guarantee synchronous correspondence between the tactile responses and the reference force during dynamic loading.

Step 1: Noise Suppression.

The tactile signals were acquired using the proposed hybrid-frequency sampling strategy (with effective rates ranging from 100 Hz to 2 kHz depending on the channel priority). For the dynamic response analysis (typically sampled at an effective rate of 1 kHz), a Savitzky–Golay digital smoothing filter was applied to mitigate random quantization noise and electromagnetic interference. A conservative window size of 30 samples (equivalent to a 30 ms time window at 1 kHz) with a polynomial order of 2 was selected. This approach effectively enhances the signal-to-noise ratio (SNR) while minimizing signal distortion and maintaining the dynamic temporal resolution required to capture rapid sliding interactions.

Step 2: Peak Extraction and Normalization.

For each sensing point, the peak response values were extracted from repeated loading trials under quantitative normal forces ranging from 0 N to 10 N. Based on the averaged peak responses, the data were normalized with respect to the peak response recorded under the maximum load (10 N), which serves as the reference value. This peak-based proportional normalization is employed to highlight the relative intensity-dependent response behavior at each sensing location while eliminating absolute sensitivity differences among different sensing units.

Step 3: Correlation Analysis with Reference Force.

For dynamic loading experiments, the normalized output of the tactile sensing system and the normalized force measured by the external reference platform are paired sample by sample. The degree of agreement between the two normalized signals is quantified using the coefficient of determination $R^{2}$, which is defined as:

(4) $$R^{2}=1-\frac{\Sigma_{k=1}^{M}{(S}_{k}-F_{k}{)}^{2}}{\Sigma_{k=1}^{M}(F_{k}-\bar{F}{)}^{2}}\;,$$

where $F_{k}$ denotes the normalized force measured by the external reference platform at sample *k*, $S_{k}$ denotes the corresponding normalized tactile output of the electronic skin system, $\bar{F}$ is the mean value of the reference force $F_{k}$, and *M* is the total number of samples. This definition corresponds to the standard statistical coefficient of determination, which represents the proportion of the variance in the reference signal that is predictable from the tactile sensor output. Unlike the Pearson correlation coefficient, which only measures linear association, this $R^{2}$ metric rigorously evaluates the goodness-of-fit by penalizing deviations from the ideal 1:1 identity line, thereby providing a robust indicator of the system’s tracking accuracy [28]. Prior to the correlation analysis, both the external reference force and the tactile sensing outputs are normalized using the maximum force recorded under the same loading condition as the normalization reference, ensuring that the two signals are compared on a consistent dimensionless scale.

Step 4: Repeatability Evaluation.

To evaluate measurement repeatability under identical normal-force loading conditions, repeated loading cycles are conducted. For each tactile channel, the peak response values obtained from multiple trials are extracted. The repeatability is quantified using the relative standard deviation (*RSD*):

(5) $$RSD=\frac{\sigma }{\mu }\times 100\%\;,$$

where *σ* and *μ* denote the standard deviation and the mean value of the peak responses, respectively. The maximum *RSD* among all evaluated channels is used as the repeatability indicator of the tactile sensing system.

All signal processing, regression analysis, and statistical calculations are performed using standard numerical analysis tools.

## 3. Results

### 3.1. Validation of Hybrid Sampling Strategy

To validate the feasibility and effectiveness of the proposed hybrid system, we conducted both logic simulation and numerical analysis prior to physical testing.

**Figure 6:**
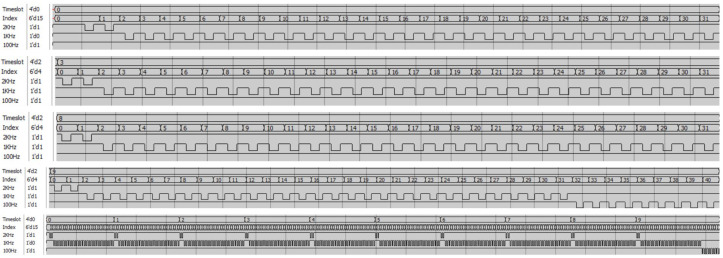
Hardware logic verification of the hybrid-frequency scheduler via ModelSim RTL simulation. The figure presents detailed waveform segments at specific time intervals (0–1 ms, 3–4 ms, 8–9 ms, and 9–10 ms) to visualize intra-frame switching, alongside a global view of the complete 0–10 ms frame (bottom panel). Row 4 (1 kHz logic) exhibits the densest switching activity, corresponding to the sequential scanning of the 30 standard-priority channels within each 1 ms slice. Row 3 (2 kHz logic) shows the distinct dual-trigger transitions for the high-priority acoustic channels, while Row 5 (100 Hz logic) reflects the sparse sub-sampling sequence for the peripheral static channels. These results confirm that the hardware scheduler deterministically executes the multi-rate polling strategy defined in the Look-Up Table (LUT). Note that the red waveforms indicate undefined states during the initialization phase.

First, a Register Transfer Level (RTL) simulation was performed using ModelSim to verify the hardware execution of the scheduler. As shown in Figure 6, the timing diagram confirms that the FPGA correctly generates the channel-selection signals. The simulation results clearly reflect the relationship between channel count and switching activity: The 1 kHz logic (Row 3) displays the most intensive signal transitions, which verifies the continuous polling of the 30 standard-priority channels (including 25 tactile, 3 acceleration, and 2 environmental sensors) occupying the majority of the time slots. The 2 kHz logic (Row 2) shows distinct activation patterns corresponding to the 2 high-priority acoustic channels, ensuring they are triggered at the correct double-rate intervals. The 100 Hz logic (Row 4) correctly executes the lower-rate scanning for the 9 peripheral channels. Crucially, this simulation demonstrates a strict one-to-one correspondence with the physical hardware operation, confirming that the actual device output matches the simulated waveforms exactly.

This validates that the Weighted Round-Robin logic is functionally correct and strictly follows the Look-Up Table configuration.

**Figure 7:**
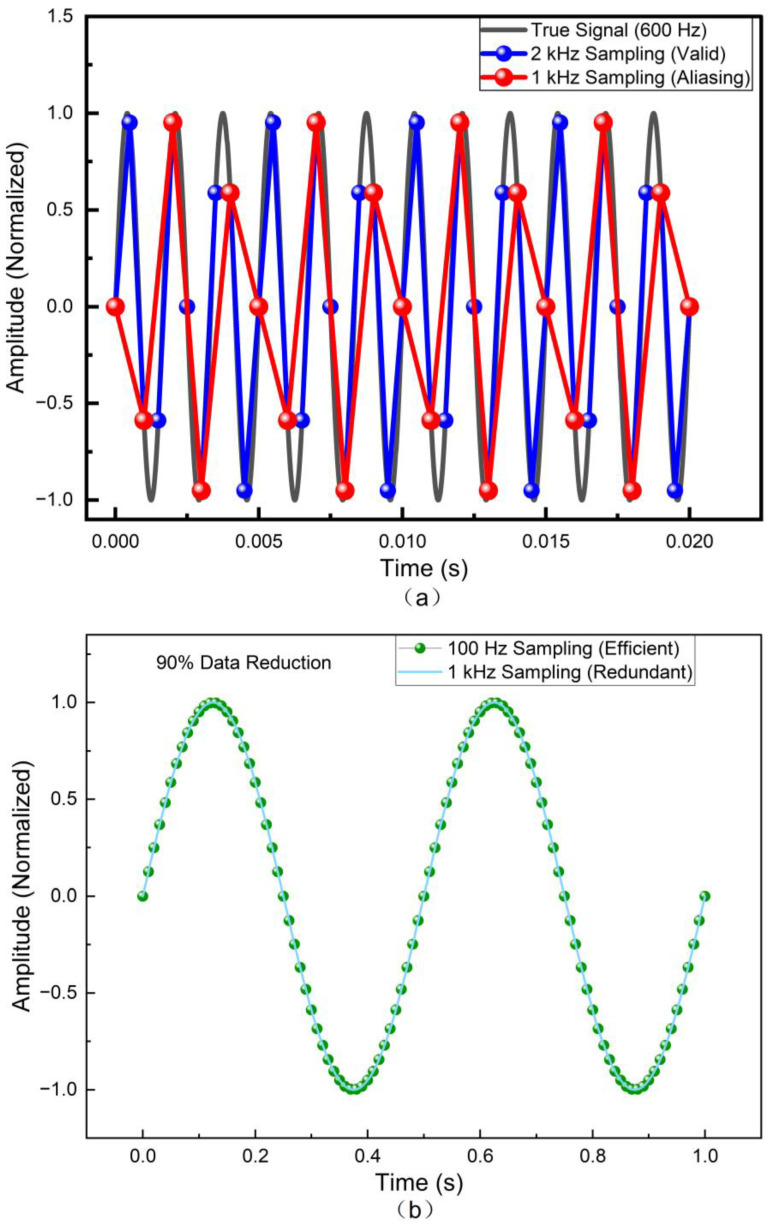
Numerical validation of the sampling rate effectiveness. (**a**) Acoustic/Texture Validation: Signal reconstruction of a 600 Hz vibration signal. The 2 kHz sampling (Blue) correctly resolves the waveform, whereas the standard 1 kHz sampling (Red) results in aliasing. (**b**) Static/Grasping Validation: Efficiency comparison for a 2 Hz quasi-static signal. The 100 Hz sampling (Green) maintains identical fidelity to the 1 kHz sampling (Light Blue) but achieves a 90% reduction in data redundancy.

Furthermore, we analyzed the signal reconstruction performance to ensure the chosen frequencies are effective for their respective tasks (Figure 7).

For high-frequency acoustic tasks, Figure 7a demonstrates that a standard 1 kHz rate fails to satisfy the Nyquist theorem for texture-induced micro-vibrations (e.g., 600 Hz), resulting in significant aliasing artifacts (falsely showing a 400 Hz slow wave). In contrast, the dedicated 2 kHz slots effectively resolve the 12-cycle waveform, proving their necessity for fine texture recognition.

Conversely, for quasi-static monitoring, Figure 7b shows that downsampling static interaction signals (2 Hz) to 100 Hz preserves 99% of the signal information compared to the 1 kHz baseline. This validates that the hybrid strategy effectively reduces the data bandwidth load by 90% for non-critical zones without compromising measurement accuracy.

### 3.2. Mechanical Flexibility and Durability Characterization

Since the tactile array is fabricated on a flexible FPC substrate (Polyimide), it is designed to conform to curved surfaces. To validate the mechanical reliability of the circuit system under long-term deformation, we conducted a cyclic bending test.

The sensor array was subjected to periodic bending operations with a bending radius of approximately 30 mm. The baseline output voltages of four representative sensing channels (CH2, CH4, CH7, CH14) were recorded under unloaded conditions every 100 bending cycles.

**Figure 8:**
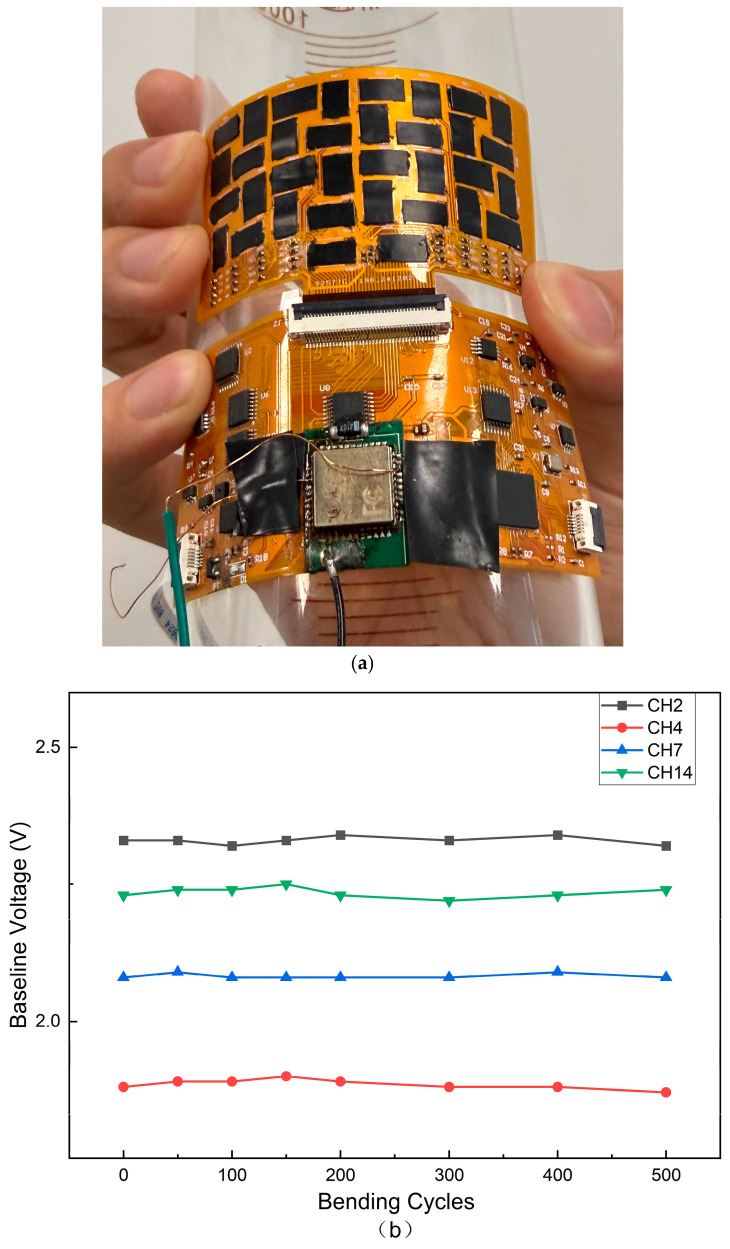
Mechanical durability evaluation of the flexible sensor array. (**a**) Optical photograph showing the flexible tactile array under bending deformation. (**b**) Baseline voltage variations in four representative channels over 500 bending cycles. The results show negligible signal drift, confirming the mechanical robustness of the circuit system.

As shown in Figure 8, the quantitative results in Figure 8b demonstrate that although the initial baseline voltages differ among channels due to inherent material variations and assembly pre-loading, the electrical response of each channel exhibits remarkable stability throughout the test. After 500 bending cycles, the fluctuation in baseline voltage for all tested channels is negligible (standard deviation < 0.05 V), with no signs of open circuits or signal drift. This result confirms that the flexible FPC interconnects and the conductive adhesive interfaces maintain reliable electrical contact even under repeated mechanical deformation.

### 3.3. Responses Under Normal-Force Loading at Different Intensities

To comprehensively evaluate the spatial uniformity and response consistency of the sensor array, the static loading experiments were significantly expanded. Instead of limited sampling, six representative sensing units were selected from distinct spatial locations to cover the typical mechanical boundary conditions of the flexible PCB. Specifically, two units were selected from the geometric center (CH11, CH15), two from the lateral edges (CH3, CH9), and two from the corners (CH1, CH4).

**Figure 9:**
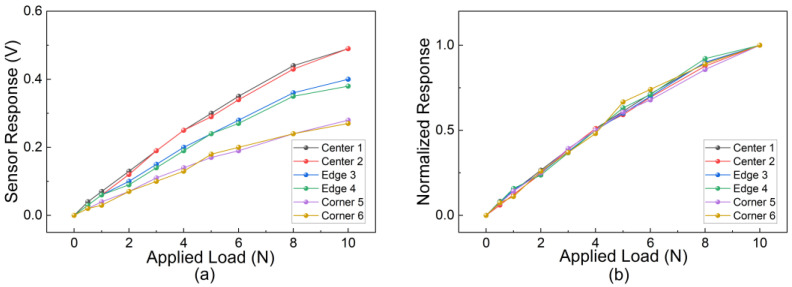
Static response characteristics of the tactile sensor array at six representative locations selected from the center, edge, and corner regions. (**a**) Raw voltage response (change from baseline) versus applied normal force (0–10 N), illustrating the variation in absolute sensitivity (Center > Edge > Corner) caused by mechanical boundary conditions. (**b**) Normalized response curves, demonstrating highly consistent linearity and response trends across the entire array regardless of spatial location.

Quantitative normal loads ranging from 0 to 10 N were applied using a precision force-loading platform. The results are presented in Figure 9. As shown in Figure 9a, the raw voltage responses of all six channels exhibit a consistent monotonic increase with applied force. It is observed that the absolute sensitivity varies with location: the center units show the highest response (~0.49 V at 10 N), while the corner units show the lowest (~0.28 V at 10 N). This gradient is consistent with the mechanical deformation theory of clamped flexible plates, where boundary constraints at the corners increase structural stiffness. However, as demonstrated in Figure 9b, after normalizing the data against the peak response, the curves from all six locations overlap significantly. This confirms that despite the mechanical variations in absolute amplitude, the linearity and response behavior of the sensing units remain highly consistent across the entire sensing surface.

Based on the quantitative calibration data presented in Figure 9a, the key performance indicators of the sensor units were extracted. The sensing units exhibit a broad effective measurement range of 0–10 N, covering the typical force levels required for tactile interaction. Within the linear response region (0–5 N), the sensing units at the geometric center demonstrate a high sensitivity of approximately 60.0 mV/N. Quantitative analysis of the calibration curves confirms that the sensor output exhibits highly linear characteristics within this primary working region (0–5 N), which validates the constant sensitivity metric derived above. While the sensitivity decreases slightly to ~36.0 mV/N at the clamped corners due to mechanical stiffening, the measurement range remains consistent across the array. These indicators confirm that the proposed sensor system possesses sufficient sensitivity and dynamic range for detecting both gentle touches and firm grasping forces.

### 3.4. Repeatability Under Repeated Normal-Force Loading

To strictly evaluate the repeatability and dynamic stability of the tactile sensor system, cyclic loading tests were conducted using a programmable force-loading platform. A sensing unit at the geometric center was subjected to controlled normal forces of 2 N and 4 N, which were applied and released consecutively for 20 cycles with a dwell time of approximately 1 s for each loading phase.

**Figure 10:**
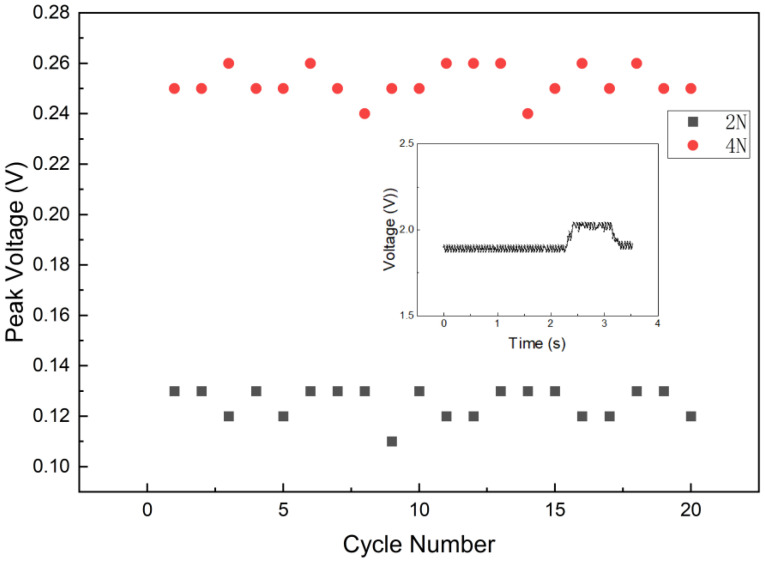
Repeatability test results under cyclic normal loads of 2 N and 4 N. The main plot tracks the peak voltage change (ΔV) for 20 consecutive cycles, demonstrating high stability with RSD values of 4.86% (2 N) and 2.53% (4 N). The inset displays the raw transient waveform for a single representative cycle under the 2 N load, showing the clear step response and signal quality.

The experimental results are presented in Figure 10. The main plot displays the cycle-to-cycle variation in the peak response voltage (ΔV) across the 20 test cycles for both load levels. It can be observed that the sensor output remains highly stable with minimal fluctuation under both low (2 N) and medium (4 N) loading conditions. To quantify this stability, the relative standard deviation (RSD) of the peak responses was calculated. The RSD values are 4.86% for the 2 N load and 2.53% for the 4 N load, indicating excellent repeatability and consistent electrical characteristics over continuous operation.

Furthermore, the inset of Figure 10 illustrates the transient raw voltage waveform of a single representative cycle under 2 N loading. The waveform exhibits a distinct step response with a stable baseline and rapid recovery times during the loading and unloading phases. The signal indicates no significant hysteresis or drift, verifying that the viscoelastic piezoresistive material possesses sufficient resilience for real-time tactile sensing applications.

### 3.5. Spatiotemporal Dynamic Responses During Sliding Interaction

To systematically evaluate the spatiotemporal tracking capability of the tactile sensing system, dynamic sliding experiments were conducted using the X–Y motion platform. A silicone finger was controlled to slide across the sensor array surface at a constant speed of 10 mm/s. This specific speed was selected to ensure a sufficient contact duration for each sensing unit and to clearly resolve the temporal evolution of the tactile stimuli.

**Figure 11:**
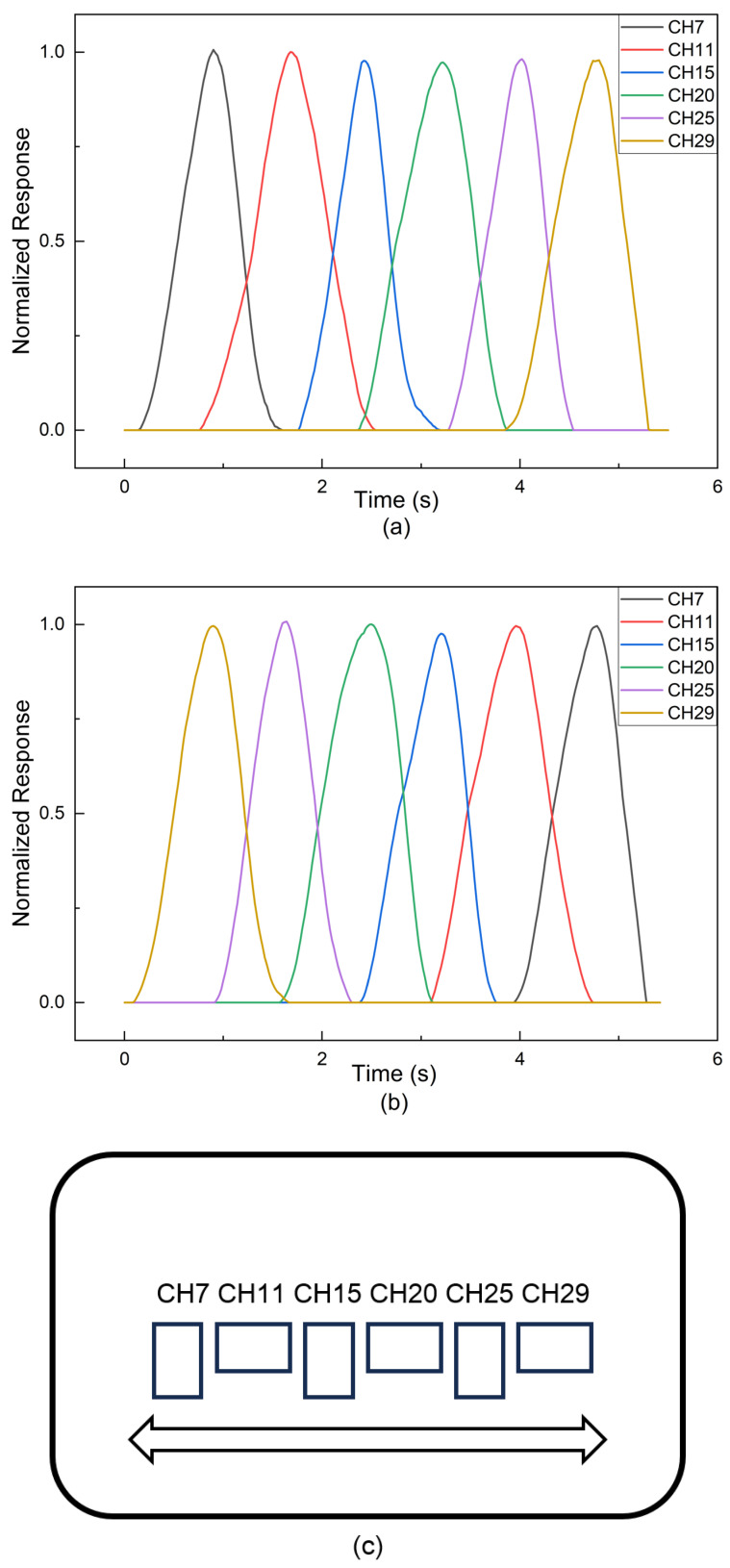
Spatiotemporal dynamic responses of the tactile array during bidirectional sliding at a speed of 10 mm/s. The normalized time-domain responses of six representative sensing units in the central region (CH7, CH11, CH15, CH20, CH25, CH29) are displayed. (**a**) Response curves during forward stroking, showing a clear sequential activation order (CH7 → CH29) with distinct temporal intervals. (**b**) Response curves during reverse stroking, demonstrating the inverted activation sequence (CH29 → CH7). (**c**) Schematic diagram illustrating the spatial location of the six selected sensing units (highlighted) and the bidirectional sliding path across the central region of the array.

The dynamic responses were investigated under bidirectional sliding paths across a representative row of sensing units in the central region (CH7, CH11, CH15, CH20, CH25, and CH29). The spatial arrangement of these selected units is illustrated in Figure 11c. The raw signals were processed using the Savitzky–Golay smoothing filter described in Section 2.5, followed by normalization to highlight the activation sequence. It is worth noting that the raw peak voltage changes (ΔV) across the tested sensing units were highly consistent (approximately 0.25 V under 4 N loading), reflecting the uniform sensitivity of the sensor array.

As shown in Figure 11a (forward stroking), the sensing units exhibit a distinct sequential activation pattern (CH7 → CH11 → … → CH29). The normalized response profiles display clearly resolved peaks corresponding to the movement of the contact point. Meanwhile, the smooth signal transitions and partial overlap between adjacent channels effectively reflect the finite contact area of the silicone finger bridging the sensing units.

Furthermore, Figure 11b displays the response under reverse stroking. The activation sequence is effectively inverted (CH29 → CH25 → … → CH7), while the waveform profiles remain consistent with the forward case. This high degree of symmetry demonstrates that the sensor array enables reliable tracking of dynamic trajectories in bidirectional paths, with minimal directional dependency.

### 3.6. Dynamic Force Tracking and Error Analysis

To further validate the sensor’s ability to track continuous, time-varying mechanical stimuli without hysteresis, a random dynamic loading test was conducted using the closed-loop electromagnet platform.

**Figure 12:**
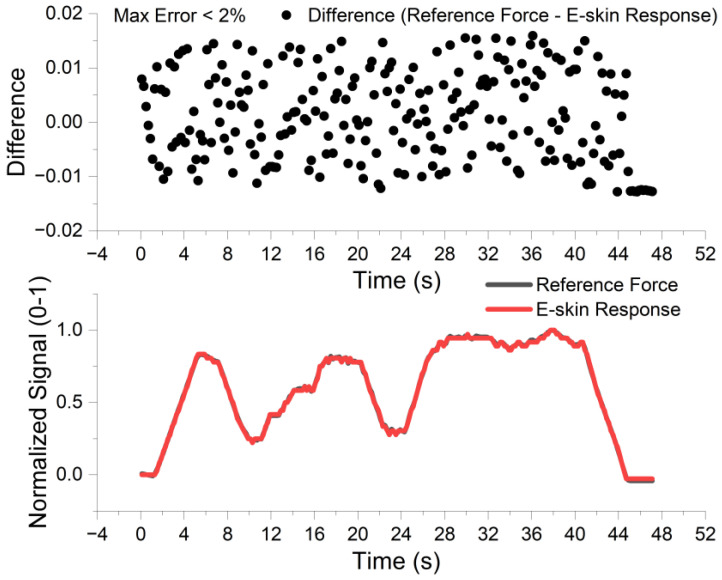
Evaluation of dynamic force tracking capability. (**Bottom**) Real-time comparison between the E-skin response (red curve) and the standard reference force (black curve) under random loading conditions. Note that both signals were normalized to the range [0, 1] for direct comparison. (**Top**) The tracking error distribution (calculated as Reference − E-skin). The results indicate a high tracking fidelity with a maximum relative deviation of less than 0.02 (<2%) throughout the testing period.

As illustrated in Figure 12, the electromagnet generated a complex force profile with varying amplitudes and durations. The integrated load cell recorded the ground truth force, while the E-skin array captured the real-time voltage response. To enable a quantitative comparison between the physical force data (measured in Newtons) and the electrical signal (measured in Volts), min-max normalization was applied to map both datasets to the dimensionless range of [0, 1].

The visual overlay in the bottom panel of Figure 12 demonstrates that the E-skin signal (red curve) tightly follows the reference force trajectory (black curve) with negligible delay. Furthermore, the tracking error analysis (top panel) reveals that the difference between the normalized sensor output and the reference load remains within a narrow band of ±0.02. This corresponds to a relative error of less than 2% of the full scale, confirming the system’s high linearity and rapid dynamic response suitable for precise tactile feedback tasks. In addition to the error analysis, the statistical correlation analysis yields a coefficient of determination of approximately 0.98, indicating that the sensor system can faithfully track the complex temporal variations in the reference force with high fidelity.

## 4. Discussion

The proposed multi-unit tactile sensing system demonstrates stable and reliable performance under dynamic loading conditions, confirming the effectiveness of combining a flexible tactile array with an FPGA-based hybrid-frequency acquisition architecture for dynamic tactile sensing. In the implemented system, most tactile channels are sampled at 1 kHz, while two channels operate at 2 kHz and nine channels are sampled at 100 Hz, enabling different sensing units to meet their respective dynamic performance requirements. By assigning higher sampling rates only to channels requiring fast dynamic response and lower rates to less critical channels, the hybrid-frequency strategy effectively reduces system complexity and overall data bandwidth, while still providing sufficient temporal resolution to accurately capture fast-changing and transient contact processes.

The overall system adopts a multi-rate coordinated acquisition architecture, including a 1 kHz subsystem for tactile, acceleration, and environmental sensing, a 2 kHz acoustic channel, and a 100 Hz quasi-static force sensing subsystem. However, it should be emphasized that the experimental investigations in this work focus exclusively on the dynamic performance of the tactile and force sensing channels, and no multi-modal experimental validation was conducted for the acoustic, acceleration, or environmental sensing channels. The multi-rate architecture is presented as a system-level design feature intended to support future extensions toward multi-modal tactile sensing, while the present study concentrates on the dynamic response characteristics of the tactile array itself.

In addition to sensing performance, the mechanical reliability of the system is critical for wearable applications. The cyclic bending results (Figure 8) confirm that the FPC-based interconnects and conductive adhesive interfaces maintain stable electrical contact (baseline drift < 0.05 V) even under repeated deformation, verifying the system’s suitability for conformable integration.

Dynamic loading experiments demonstrate that the constructed tactile array system can clearly distinguish different types of tactile stimuli, including gentle tapping, moderate pressing, and firm contact. The responses of the sensing channels exhibit consistent intensity-dependent trends, indicating that the multi-unit tactile array maintains stable and consistent force-response characteristics under dynamic loading conditions. The achieved repeatability better than 4.9% further reflects the overall measurement stability of the system during repeated dynamic loading cycles. These results indicate that the system can effectively capture dynamic tactile interactions characterized by the time-varying and spatially moving distribution of normal contact force across the tactile array.

The strong linear correlation (R^2^ ≈ 0.98) between the tactile array outputs and the external reference forces provides system-level validation of the platform’s capability to accurately represent external mechanical stimuli. This correlation reflects the coordinated performance of the complete sensing chain, including the flexible tactile array, analog front-end circuits, multiplexing behavior, FPGA-based timing control, and data transmission, rather than the behavior of individual sensing elements in isolation. Such system-level validation is particularly important for practical dynamic tactile applications, where high-fidelity real-time acquisition is required [29,30].

Compared with many previously reported flexible tactile systems that rely on low-frequency acquisition (typically <100 Hz) or single-point static indentation tests, the proposed system achieves a substantially higher effective sampling rate of 1 kHz across the dynamic sensing zone (25 channels) and 36.9 kHz aggregate throughput. This performance corresponds to an aggregate system throughput of approximately 36.9 kHz, which is rigorously derived from the deterministic global frame structure: within each 10 ms cycle, the system transmits 369 data packets (comprising 360 high/standard-priority samples and 9 low-priority samples, including protocol overheads). Furthermore, the hardware-level execution of this 1 kHz synchronous scanning loop is explicitly verified by the RTL simulation logic presented in Figure 6. This capability enables reliable tracking of rapid and transient tactile events as well as accurate capture of spatiotemporal dynamic patterns (e.g., finger stroking). It is worth noting that this 1 kHz effective sampling rate is a deterministic hardware constraint enforced by the FPGA’s Weighted Round-Robin scheduler, rather than a software-dependent theoretical maximum. As verified by the RTL simulation in Section 3.1 (Figure 6), the acquisition system rigorously executes the full channel-scanning loop (covering all 34 sensing units) within every 1 ms global frame, regardless of the number of active contact points. Consequently, even in application scenarios where only a subset of sensors is mechanically activated (e.g., the finger stroking demonstration in Figure 11), the system continues to sample the entire array at full speed, ensuring that any sudden or multi-point contact events are captured with zero latency. A detailed performance comparison with representative state-of-the-art flexible piezoresistive tactile arrays is summarized in Table 2.

As shown in Table 2, while some systems offer higher single-sensor bandwidth or larger array scales, most suffer from limited synchronized sampling rates and less comprehensive dynamic validation under moving contact conditions. Although fully parallel-ADC architectures can theoretically eliminate channel-switching delay and achieve perfect synchronization, they often involve significantly higher hardware cost, power consumption, and system complexity, which limits their suitability for compact, embedded, or wearable implementations. In contrast, the acquisition strategy adopted in this work, combined with deterministic FPGA-based global-frame timing control, provides an effective engineering trade-off that maintains high dynamic performance while ensuring hardware efficiency and scalability.

Furthermore, many existing studies on flexible tactile arrays primarily emphasize material properties or device-level sensitivity, with limited attention to complete system-level validation under dynamic conditions. In contrast, this work evaluates the tactile array, analog front-end, multiplexing structure, timing control, and cross-validation with external reference forces in an integrated manner under realistic dynamic loading. This system-level evaluation highlights the robustness and practical applicability of the proposed architecture for real-world tactile interaction scenarios [31,32].

Overall, the present study verifies the reliability of a synchronized multi-unit tactile sensing platform for dynamic tactile acquisition. Although the multi-rate architecture of the system was not fully exploited for multi-modal experimental validation in this work, it provides a feasible hardware basis for future studies on multi-modal tactile perception, intelligent interaction, and sensory data fusion [33,34].

## 5. Conclusions

This work presents and validates a synchronized multi-unit tactile sensing system based on a flexible piezoresistive sensor array. An FPGA-controlled hybrid-frequency acquisition architecture is employed to synchronously sample the sensor array using a hybrid strategy, achieving 1 kHz sampling for dynamic interaction zones. Hardware logic verification confirms the precise execution of the scheduling timing, and mechanical durability tests demonstrate the system’s robustness against bending.

Experimental results demonstrate that the proposed system can stably acquire dynamic tactile responses and distinguish different loading intensities, with good measurement repeatability (better than 4.9%) and strong correlation with external reference forces (R^2^ ≈ 0.98). These results further confirm that the proposed system can reliably capture dynamic tactile interactions characterized by time-varying and spatially moving distributions of normal contact force across the tactile array.

## Figures and Tables

**Table 1:** Look-Up Table (LUT) configuration for the Weighted Round-Robin scheduler, illustrating the mapping of time slots to multimodal sensing channels.

Time Slot Index (FPGA Logic)	Target Modality	Physical Channels	Sampling Strategy
0, 1	Acoustic	CH35, CH36 (Acoustic/Vibration)	High (2 kHz) (Mapped to 2 slots)
2~14	Tactile (Dynamic)	CH1~CH13	Standard (1 kHz)
15, 16	Acoustic	CH35, CH36 (Acoustic/Vibration)	High (2 kHz) (Re-entry)
17~29	Tactile (Dynamic)	CH14~CH25	Standard (1 kHz)
30~34	Inertial & Env.	X/Y/Z Accel, Temp, Humidity	Standard (1 kHz)
35~43	Tactile (Static)	CH26~CH34 (Peripheral)	Low (100 Hz) (Sub-sampled)

**Table 2:** Comparison of the proposed synchronized multi-unit tactile sensing system with representative state-of-the-art flexible piezoresistive tactile arrays.

System/Research	Array Scale	Sampling Rate/Frequency Range (Hz)	Sensitivity	Repeatability/Durability	Linearity/Correlation
This Work	34 channels	1 kHz (dynamic zone)	~60 mV/N (0–5 N)	RSD < 4.9%; 500 bending cycles	R^2^ ≈ 0.98
Ref. [9] (Bao et al., 2023)	Not specified (piezoresistive array for robotic grasping)	Not specified (but involves time and frequency domain characteristics)	High (piezoresistive, with correlation coefficients)	High repeatability (RSD < 4.9%)	Strong linear correlation (R^2^ ≈ 0.98)
Ref. [20] (Zhu et al., 2020)	Multi-channel	Not specified	High (10.89 ± 0.5 mV/kPa in 80–230 kPa range)	Superior mechanical durability (>8500 working cycles)	High (dynamic response)
Ref. [21] (Zhu et al., 2021)	Not specified	Not specified (but limited by low-frequency AC currents)	High (gauge factor GF = 17.4 in 0–0.6% strain)	Good fatigue resistance (over 1000 cycles)	High (bending/torsion detection)
Ref. [8] (Bandodkar et al., 2020)	Epidermal multi-channel	Not specified (but involves signal conditioning)	High (physiological signal monitoring)	Superior mechanical durability (>8500 working cycles)	Strong (high physiological signal correlation)
Ref. [17] (Bai et al., 2023)	Multi-dimensional wearable	Not specified (but involves stimuli-responsive characteristics)	High (strain sensing)	High softness and durability	High (strain, temperature, humidity sensing)
Ref. [24] (Zhuang et al., 2024)	High-density	Not specified (but involves signal processing and analysis)	High (biosignal sensing)	High softness and durability	High (signal analysis)

## Data Availability

The data presented in this study are available on request from the corresponding author.
